# Vertical versus transverse abdominal incision in placental abruption: a propensity score-matched study on the trade-off between speed and maternal-neonatal safety

**DOI:** 10.3389/fmed.2026.1804028

**Published:** 2026-05-29

**Authors:** Kai Sun Zhao, Xia Chu Wei, Chun Lan Yuan, Shi Mei He, Mei Rong He, Ni Bei, Jie Cheng Bi, Jian Chun Huang

**Affiliations:** Department of Obstetrics, The Second Nanning People’s Hospital, The Third Affiliated Hospital of Guangxi Medical University, Nanning, Guangxi, China

**Keywords:** decision-to-delivery interval, emergency cesarean section, maternal-fetal outcomes, midline vertical incision, placental abruption, propensity score matching

## Abstract

**Objective:**

To compare maternal and neonatal outcomes between midline vertical and transverse abdominal incisions during emergency cesarean delivery for placental abruption.

**Methods:**

In this retrospective cohort study, 152 patients were included. Propensity score matching (PSM) was used to balance baseline characteristics, resulting in 106 well-matched patients (41 vertical, 65 transverse). Surgical metrics, laboratory parameters, and a composite adverse outcome were compared. Independent predictors were identified via multivariate logistic regression.

**Results:**

After PSM, the vertical group had a shorter median decision-to-delivery interval (DDI: 17.0 vs. 28.0 min, *p* < 0.001) but a significantly higher incidence of the composite adverse outcome (75.6% vs. 32.3%, *p* < 0.001), surgical site complications (29.3% vs. 0%), preterm birth, neonatal asphyxia, and NICU admission. Vertical incision (adjusted OR = 3.59, 95% CI 1.04–12.38), earlier gestational age, lower preoperative fibrinogen, and shorter DDI were independent risk factors.

**Conclusion:**

In this PSM analysis, vertical incision was associated with faster delivery but also with significantly worse composite maternal-neonatal outcomes. These findings underscore a trade-off between speed and morbidity. However, confounding by indication may influence this association. Incision selection in this setting warrants individualized assessment.

## Introduction

Placental abruption is a critical obstetric emergency often necessitating emergency cesarean delivery, where the decision-to-delivery interval (DDI) is a key modifiable factor for neonatal outcome (1, 2). The choice of abdominal incision–midline vertical versus transverse–represents a fundamental surgical decision that influences both the speed of delivery and postoperative recovery. Standardized cesarean techniques have been demonstrated to correlate with improved patient outcomes (3, 4). While vertical incisions are traditionally considered to allow quicker abdominal entry, they are associated with greater tissue trauma and higher risks of wound complications (5). This trade-off is particularly relevant in placental abruption, a condition frequently complicated by coagulopathy and impaired healing (1, 6). However, high-quality evidence directly comparing these incision types in the specific, high-risk context of placental abruption remains scarce. Existing studies are mostly retrospective and involve heterogeneous populations, with limited control for baseline confounding (7–9). The net effect of incision choice on comprehensive maternal-neonatal outcomes in this volatile setting is therefore unclear.

To address this evidence gap, we conducted a retrospective cohort study utilizing propensity score matching (PSM) to compare maternal-neonatal outcomes between midline vertical and transverse abdominal incisions exclusively in patients with placental abruption. By minimizing baseline confounding through PSM, this study aims to provide a more robust evaluation of the net effect of incision type. Furthermore, we sought to identify independent risk factors for a composite adverse outcome to inform risk stratification and individualized surgical decision-making in this critical setting.

## Materials and methods

### Study design and ethical approval

This was a single-center, retrospective cohort study conducted in accordance with the Declaration of Helsinki. The study protocol received approval from the Institutional Ethics Committee of Nanning Second People’s Hospital, which waived the requirement for informed consent due to the retrospective design.

The need for written informed consent was waived by the Ethics Committee due to the retrospective, observational nature of this study. The analysis utilized exclusively anonymized clinical and laboratory data that were originally collected for routine diagnosis and treatment purposes. The use of this de-identified data for research posed no more than minimal risk to the patients, and it was impracticable to obtain consent from all individuals given the large cohort and the fact that data were fully anonymized, preventing re-contact. All data were handled in strict confidentiality and in compliance with the principles of the Declaration of Helsinki.

### Compliance with EQUATOR network guidelines

This study is reported in accordance with the Strengthening the Reporting of Observational Studies in Epidemiology (STROBE) statement for observational research. A completed STROBE checklist is provided as Supplementary material.

### Study population and data collection

We retrospectively identified 152 patients who underwent emergency cesarean delivery for placental abruption at our institution between January 2020 and December 2024.

### Inclusion and exclusion criteria

Inclusion criteria comprised: (1) singleton pregnancy; (2) gestational age ≥ 28 weeks at delivery; (3) emergency cesarean section for placental abruption; (4) complete surgical documentation specifying the abdominal incision type (midline vertical or transverse); and (5) availability of complete clinical and laboratory data.

**FIGURE 1:**
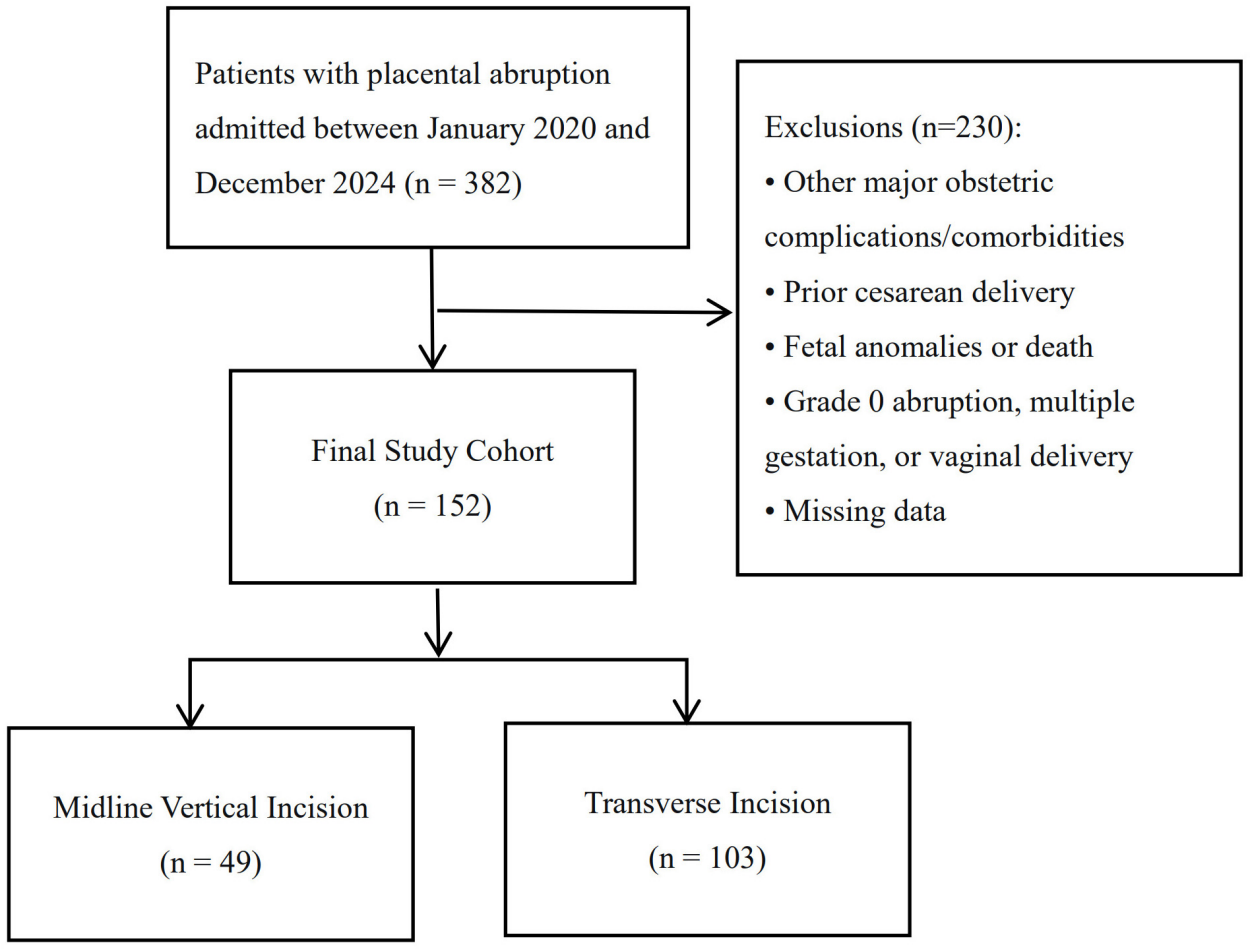
Flowchart of patient screening and enrollment.

Exclusion criteria were: (1) co-existing major obstetric complications (e.g., placenta accreta spectrum, placenta previa, uterine rupture); (2) significant medical or surgical comorbidities (e.g., severe cardiopulmonary dysfunction, uncontrolled chronic hypertension or diabetes mellitus); (3) prior cesarean delivery; (4) major fetal congenital anomalies or chromosomal abnormalities; (5) intrauterine fetal death at admission; (6) diagnosis of grade 0 placental abruption (Grade 0 placental abruption was defined as a retrospective diagnosis based on the finding of a retroplacental clot upon postpartum placental inspection, in the absence of any antepartum clinical symptoms (e.g., vaginal bleeding, abdominal pain) or fetal heart rate abnormalities); (7) multiple gestation; (8) vaginal delivery; or (9) missing medical records or essential information. The patient selection flowchart is presented in Figure 1.

### Study groups

Patients were categorized into two groups based on the abdominal incision used: the Midline Vertical Incision group and the Transverse Incision group.

### Clarification on incision selection and surgical context

During the study period, all included cesarean deliveries for placental abruption were managed as Category I (most urgent) emergency procedures. There was no mandatory institutional protocol dictating the choice of abdominal incision. The decision between a midline vertical or transverse incision was ultimately at the discretion of the attending obstetrician, based on their clinical judgment and real-time assessment of individual patient factors such as the acuity of fetal distress, maternal hemodynamic stability, and the anticipated need for rapid delivery or extended surgery (e.g., potential hysterectomy). All primary surgeons were credentialed attending obstetricians (at the rank of attending or associate chief physician) with over 10 years of clinical practice, and a total of 9 attending obstetricians were involved in performing the procedures across the entire study cohort, This ensuring a high and consistent level of clinical judgment and surgical proficiency across all cases.

### Outcome measures

#### Primary outcome

The primary endpoint was a predefined composite adverse outcome, comprising any of the following: intra- or postoperative blood transfusion, surgical site complications (redness/liquefaction), postpartum ICU admission, neonatal asphyxia, or NICU admission. A patient was considered to have reached the endpoint upon experiencing at least one of these events during hospitalization.

### Data collection

Data were retrospectively collected from the electronic medical records, including:

Maternal Baseline Characteristics: Age, gravidity, parity, pre-pregnancy body mass index (BMI), antenatal BMI.

Surgical Parameters: DDI, incision-to-delivery interval, total operative time, estimated blood loss (determined by the combined volumetric and gravimetric method, i.e., measuring blood in the suction canister after accounting for amniotic fluid plus weighing blood-soaked sponges and drapes), anesthesia type, uterine packing, ligation of the uterine artery ascending branch.

Laboratory Parameters: Pre- and postoperative hemoglobin (Hb), white blood cell count (WBC), neutrophil percentage (NEU%), platelet count (PLT), preoperative fibrinogen (FIB).

Maternal Outcomes: Length of postoperative hospitalization, duration of postoperative antibiotic use, hypertensive disorders of pregnancy (HDP), postpartum hemorrhage (PPH), perioperative blood transfusion, postoperative fever, surgical site complications, ICU admission.

Neonatal Outcomes: Gestational age at delivery, preterm birth, birth weight, neonatal asphyxia, NICU admission.

### Sample size considerations

As a retrospective study, no formal *a priori* sample size calculation was performed. The sample size was determined by the availability of eligible cases during the study period. While the matched cohort size (*n* = 106) is moderate, the observed effect sizes for the primary outcome were substantial (e.g., composite adverse outcome: 75.6% vs. 32.3%), and the use of propensity score matching aimed to maximize the validity of comparisons within the available data. The study’s findings should be interpreted in the context of its sample size and design.

### Propensity score matching methodology

**FIGURE 2:**
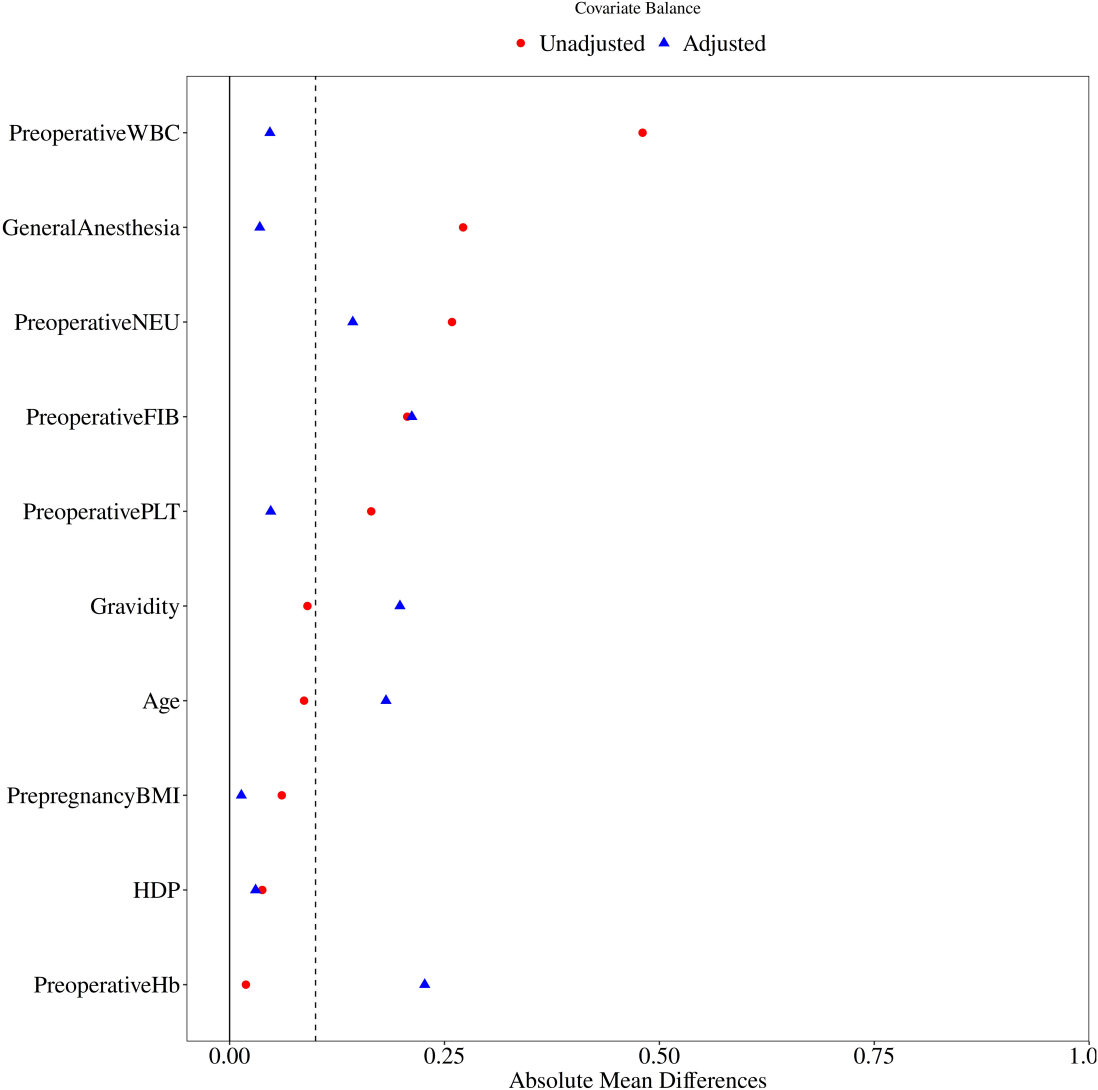
Standardized mean difference (SMD) plot.

**FIGURE 3:**
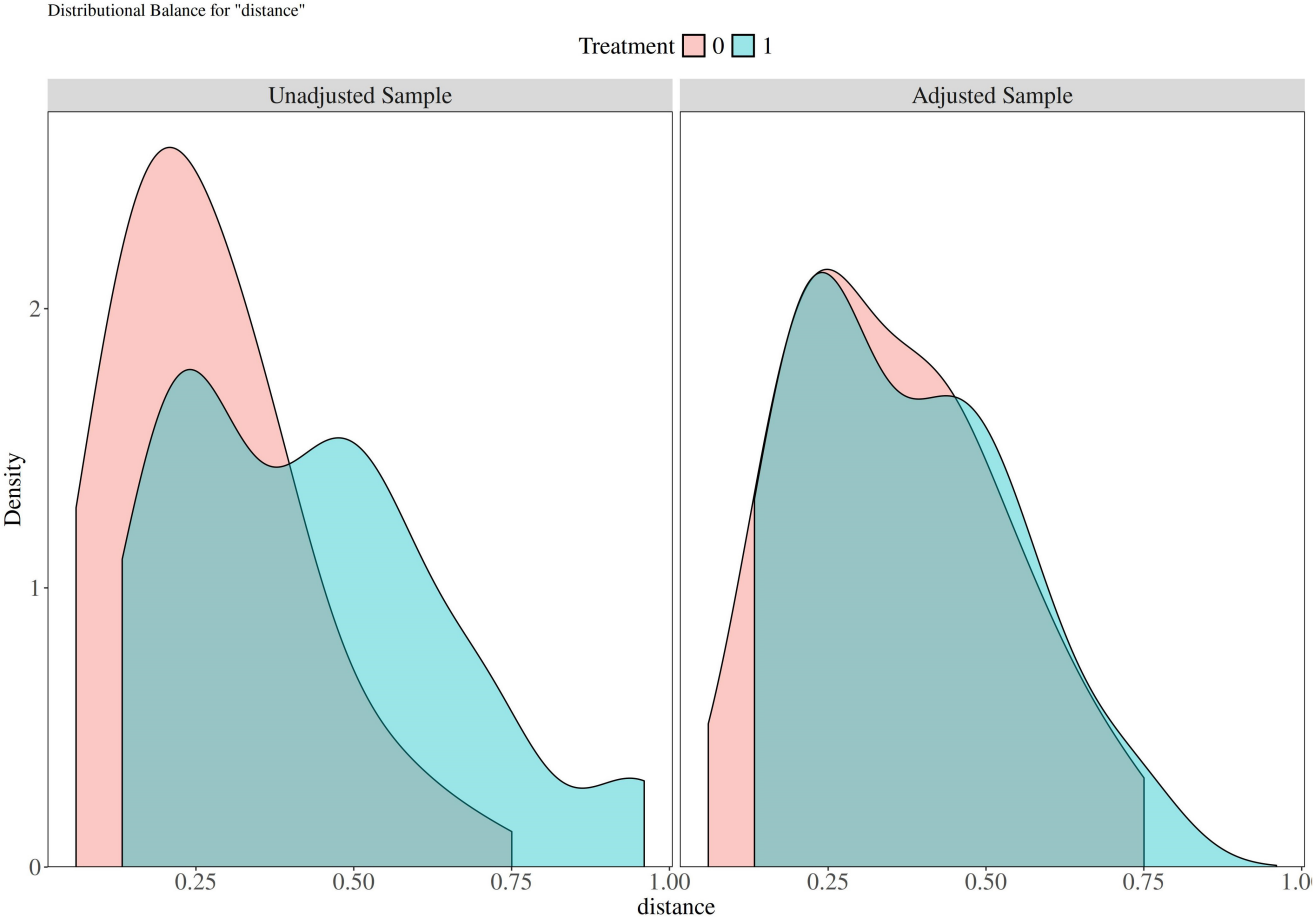
Density plot.

To mitigate baseline confounding, we performed PSM. The propensity score, representing the probability of receiving a midline vertical incision, was estimated using a logistic regression model that included the following pre-specified covariates: age, pre-pregnancy BMI, HDP, anesthesia type, gravidity, preoperative FIB, preoperative Hb, PLT, preoperative WBC, and preoperative NEU%. These variables were selected *a priori* based on clinical knowledge and literature as potential confounders influencing both incision choice and outcomes. A 1:2 nearest-neighbor matching algorithm with a caliper set to 0.2 standard deviations of the propensity score was employed. Balance between groups before and after PSM was assessed using the standardized mean difference (SMD), where an SMD < 0.1 indicates negligible imbalance. The matching procedure yielded a well-balanced cohort of 106 patients (41 midline vertical, 65 transverse incision) for primary analysis. Balance diagnostics are presented in Figure 2 (SMD plot) and Figure 3 (density plot). Although minor residual imbalances (SMD 0.1–0.2) persisted for six covariates (HDP, preoperative WBC, NEU%, Hb, PLT, FIB), the overall comparability was substantially improved and deemed acceptable for comparative analysis.

### Statistical analysis

In the matched cohort, continuous variables are presented as mean ± standard deviation or median (interquartile range) based on distribution, and were compared using independent-sample *t*-tests or Mann-Whitney U tests, respectively. Categorical variables are presented as number (percentage) and were compared using Chi-square or Fisher’s exact tests, as appropriate. To identify independent risk factors for the composite adverse outcome, univariate logistic regression was first performed. Variables with *P* < 0.10 in univariate analysis were entered into a multivariate logistic regression model with bidirectional stepwise selection. Odds ratios (ORs) with 95% confidence intervals (CIs) were calculated. Multicollinearity was assessed prior to multivariate analysis using variance inflation factors (VIF), with VIF > 5 indicating severe collinearity. To further evaluate feature importance and the direction of contributions in the prediction model, we performed SHAP (SHapley Additive exPlanations) analysis based on the same set of predictor variables, and generated a beeswarm plot to visualize the impact of each variable. The discriminatory performance of the multivariate model and key individual predictors for the composite outcome was evaluated using receiver operating characteristic (ROC) curve analysis, reporting the area under the curve (AUC). The optimal cutoff, sensitivity, and specificity were determined by maximizing Youden’s index. All statistical analyses were performed using SPSS (version 29.0), R (version 4.2.0), and the MedSci statistical platform^1^. All tests were two-sided, with *P* < 0.05 considered statistically significant.

## Results

### Comparison of baseline characteristics

After propensity score matching, baseline characteristics–including age, gravidity, parity, and pre-pregnancy and antenatal BMI–were well-balanced between the two groups, with no significant differences (*P* > 0.05) and all standardized mean differences (SMDs) < 0.1 (Table 1).

**TABLE 1:** Comparison of patient baseline characteristics before and after propensity score matching.

	Before PSM (*n* = 152)				After PSM (*n* = 106)			
	Transverse incision (*n* = 103)	Midline vertical incision (*n* = 49)	*P*	SMD	Transverse incision (*n* = 65)	Midline vertical incision (*n* = 41)	*P*	SMD
Age (years)	30.50 ± 5.53	30.02 ± 5.59	0.616	−0.087	30.12 ± 5.31	29.88 ± 5.60	0.821	−0.044
Gravidity	2.00 (1.00, 3.00)	2.00 (1.00, 3.00)	0.480	0.091	2.00 (1.00, 3.00)	3.00 (1.00, 3.00)	0.644	0.071
Parity	1.00 (1.00, 2.00)	1.00 (1.00, 2.00)	0.308	0.138	1.00 (1.00, 2.00)	1.00 (1.00, 2.00)	0.633	0.091
Pre-pregnancy BMI	20.83 (19.23, 23.29)	21.05 (19.53, 23.44)	0.675	0.061	21.62 ± 2.90	21.57 ± 2.95	0.929	−0.018
Antenatal BMI	26.12 ± 3.06	26.07 ± 3.19	0.924	−0.016	26.29 ± 3.23	26.05 ± 3.42	0.709	−0.072

BMI, body mass index; SMD, standardized mean difference; an SMD < 0.1 indicates negligible imbalance between groups after matching.

### Comparison of surgical and laboratory parameters

Following matching, the two groups were comparable with respect to the DDI, incision-to-delivery interval, rate of general anesthesia, rate of uterine artery ascending branch ligation, and multiple laboratory parameters–including pre- and postoperative NEU% and PLT, as well as preoperative FIB, Hb, and WBC (*P* > 0.05). In contrast, the midline vertical incision group demonstrated a significantly longer operative time, greater estimated blood loss, a higher rate of uterine packing, lower postoperative Hb (*P* < 0.05), as detailed in Table 2.

**TABLE 2:** Comparison of surgical and laboratory parameters before and after propensity score matching.

	Before PSM (*n* = 152)				After PSM (*n* = 106)			
	Transverse incision (*n* = 103)	Midline vertical incision (*n* = 49)	*P*	SMD	Transverse incision (*n* = 65)	Midline vertical incision (*n* = 41)	*P*	SMD
DDI (min)	38.00 (24.00, 60.00)	17.00 (9.00, 28.00)	<0.001	−1.201	28.00 (21.00, 41.00)	17.00 (9.00, 28.00)	<0.001	−0.919
Incision-to-delivery interval (min)	3.00 (2.00, 4.00)	2.00 (2.00, 3.00)	0.067	−0.759	2.00 (2.00, 4.00)	3.00 (2.00, 3.00)	0.574	−0.607
Operative time (min)	51.00 (45.00, 59.50)	63.00 (55.00, 69.00)	<0.001	0.908	51.86 ± 12.53	63.29 ± 10.80	<0.001	1.058
Estimated blood loss (ml)	400.00 (300.00, 400.00)	500.00 (400.00, 600.00)	<0.001	0.432	400.00 (300.00, 400.00)	500.00 (400.00, 600.00)	<0.001	0.402
General anesthesia, *n* (%)	54 (52.43%)	39 (79.59%)	0.001	0.674	43 (66.15%)	31 (75.61%)	0.302	0.220
Uterine packing, *n* (%)	17 (16.50%)	29 (59.18%)	<0.001	0.868	11 (16.92%)	21 (51.22%)	<0.001	0.686
Uterine artery ascending branch ligation, *n* (%)	19 (18.45%)	16 (32.65%)	0.052	0.303	13 (20.00%)	11 (26.83%)	0.413	0.154
Preoperative FIB (g/L)	4.41 (3.90, 4.97)	4.47 (3.49, 5.08)	0.610	−0.207	4.48 ± 1.00	4.25 ± 1.23	0.286	−0.190
Preoperative Hb (g/L)	118.00 (110.00, 128.00)	118.00 (106.00, 129.00)	0.776	−0.019	122.00 (111.00, 130.00)	118.00 (106.00, 128.00)	0.308	−0.131
Postoperative Hb (g/L)	108.00 (98.00, 118.00)	98.00 (82.00, 114.00)	0.004	−0.451	105.00 (98.00, 118.00)	101.00 (86.00, 114.00)	0.045	−0.331
Preoperative WBC (×10^9^/L)	9.40 (8.40, 11.00)	11.20 (9.10, 13.60)	0.002	0.480	10.39 ± 2.84	10.89 ± 2.70	0.369	0.186
Postoperative WBC (×10^9^/L)	15.10 (12.30, 18.15)	16.30 (12.90, 20.20)	0.218	0.220	15.80 ± 3.92	16.64 ± 5.61	0.406	0.150
Preoperative NEU%	0.73 ± 0.07	0.75 ± 0.09	0.084	0.259	0.72 ± 0.07	0.74 ± 0.08	0.372	0.171
Postoperative NEU%	0.82 ± 0.05	0.82 ± 0.08	0.894	0.021	0.82 ± 0.05	0.81 ± 0.07	0.448	−0.134
Preoperative PLT(×10^9^/L)	245.77 ± 63.40	233.24 ± 75.99	0.288	−0.165	242.72 ± 60.06	233.02 ± 75.62	0.466	−0.128
Postoperative PLT(×10^9^/L)	221.69 ± 56.71	199.43 ± 64.15	0.032	−0.347	217.98 ± 55.35	199.80 ± 61.10	0.117	−0.298

FIB, fibrinogen; Hb, hemoglobin; WBC, white blood cell count; NEU%, neutrophil percentage; PLT, platelet count. Operative time was measured from skin incision to skin closure. DDI, decision-to-delivery interval (time from decision for cesarean delivery to fetal delivery). Laboratory parameters were measured preoperatively (within 1–2 days before surgery) and postoperatively (on the first morning after surgery).

### Comparison of maternal and neonatal outcomes

In the matched cohort, no significant differences were found in the rates of uteroplacental stroke, postpartum fever, or PPH. However, patients in the midline vertical incision group had significantly worse outcomes across multiple measures: increased rates of preterm birth (PTB), neonatal asphyxia, NICU admission, wound complications, and the composite endpoint, as well as prolonged postoperative antibiotic use and hospitalization (*P* < 0.05; Table 3).

**TABLE 3:** Comparison of maternal and neonatal outcomes before and after propensity score matching.

	Before PSM (*n* = 152)				After PSM (*n* = 106)			
	Transverse incision (*n* = 103)	Midline vertical incision (*n* = 49)	*P*	SMD	Transverse incision (*n* = 65)	Midline vertical incision (*n* = 41)	*P*	SMD
Gestational age at delivery (days)	270.00 (256.50, 278.00)	256.00 (246.00, 270.00)	0.002	−0.451	271.00 (262.00, 278.00)	256.00 (245.00, 270.00)	<0.001	−0.541
Birth weight (g)	3000.00 (2570.00, 3200.00)	2600.00 (2300.00, 3000.00)	0.007	−0.366	3050.00 (2600.00, 3300.00)	2600.00 (2400.00, 3000.00)	0.017	−0.367
PTB, *n* (%)	28 (27.18%)	25 (51.02%)	0.004	0.477	12 (18.46%)	21 (51.22%)	<0.001	0.655
Neonatal asphyxia, *n* (%)	10 (9.71%)	24 (48.98%)	<0.001	0.786	8 (12.31%)	18 (43.90%)	<0.001	0.637
NICU admission, *n* (%)	36 (34.95%)	35 (71.43%)	<0.001	0.789	20 (30.77%)	27 (65.85%)	<0.001	0.740
HDP	15 (14.56%)	9 (18.37%)	0.548	0.098	13 (20.00%)	6 (14.63%)	0.483	−0.152
PPH	0 (0.00%)	3 (6.12%)	0.032	0.255	0 (0.00%)	3 (7.32%)	0.107	0.686
Uteroplacental apoplexy, *n* (%)	5 (4.85%)	10 (20.41%)	0.007	0.386	3 (4.62%)	7 (17.07%)	0.073	0.331
Postoperative fever, *n* (%)	12 (11.65%)	14 (28.57%)	0.010	0.375	9 (13.85%)	10 (24.39%)	0.168	0.246
Surgical site complications (redness/liquefaction), *n* (%)	2 (1.94%)	15 (30.61%)	<0.001	0.622	0 (0.00%)	12 (29.27%)	<0.001	0.643
Postoperative hospital stay (days)	5.00 (4.00, 5.00)	6.00 (5.00, 6.00)	<0.001	0.776	5.00 (4.00, 5.00)	6.00 (5.00, 6.00)	<0.001	0.758
Duration of postoperative antibiotic use (days)	1.00 (1.00, 2.00)	3.00 (2.00, 4.00)	<0.001	0.687	2.00 (1.00, 2.00)	3.00 (1.00, 4.00)	0.002	0.598
Composite adverse outcome, *n* (%)	37 (35.92%)	39 (79.59%)	<0.001	1.084	21 (32.31%)	31 (75.61%)	<0.001	1.008

PTB, preterm birth; HDP, hypertensive disorders of pregnancy; PPH, postpartum hemorrhage.

### Analysis of risk factors for the composite adverse outcome

On univariate logistic regression analysis, operative time, estimated blood loss, midline vertical incision, gestational age at delivery, preoperative fibrinogen, DDI, and preoperative hemoglobin were significantly associated with the composite adverse outcome (*P* < 0.05; Table 4).

**TABLE 4:** Results of univariate and multivariate logistic regression analyses for the composite adverse outcome.

	Univariate					Multivariate					
	β	S.E	Z	*P*	OR (95% CI)	β	S.E	Z	*P*	OR (95% CI)	VIF
Operative time (min)	0.04	0.02	2.18	0.029	1.04 (1.01–1.07)						1.544
Estimated blood loss (ml)	0.01	0.00	2.11	0.035	1.01 (1.01–1.01)						1.691
Midline vertical incision	1.87	0.45	4.16	<0.001	6.50 (2.69–15.69)	1.28	0.63	2.02	0.043	3.59 (1.04–12.38)	1.456
Gestational age at delivery (Days)	−0.10	0.02	−4.51	<0.001	0.90 (0.86–0.94)	−0.11	0.03	−3.60	<0.001	0.90 (0.85–0.95)	1.168
Preoperative FIB (g/L)	−0.53	0.21	−2.55	0.011	0.59 (0.39–0.88)	−0.78	0.38	−2.05	0.040	0.46 (0.22–0.96)	1.711
DDI (min)	−0.03	0.01	−2.75	0.006	0.97 (0.95–0.99)	−0.04	0.02	−2.32	0.020	0.96 (0.93–0.99)	1.165
Preoperative HB (g/L)	−0.04	0.01	−2.99	0.003	0.96 (0.93–0.98)	−0.03	0.02	−1.39	0.164	0.97 (0.92–1.01)	1.746

FIB, fibrinogen; Hb, hemoglobin; DDI, decision-to-delivery interval. VIF, variance inflation factor (VIF > 5 indicates severe multicollinearity).

### Multivariate analysis of risk factors for the composite adverse outcome

**FIGURE 4:**
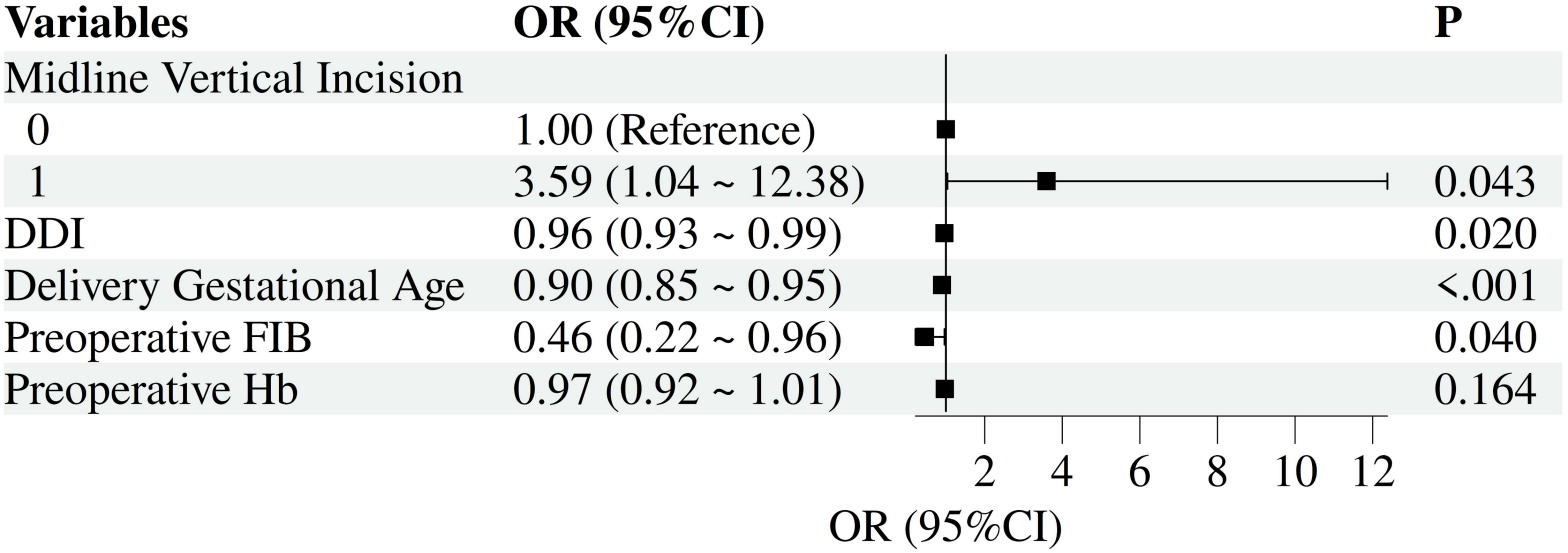
Forest plot of risk factors for the composite adverse outcome.

**FIGURE 5:**
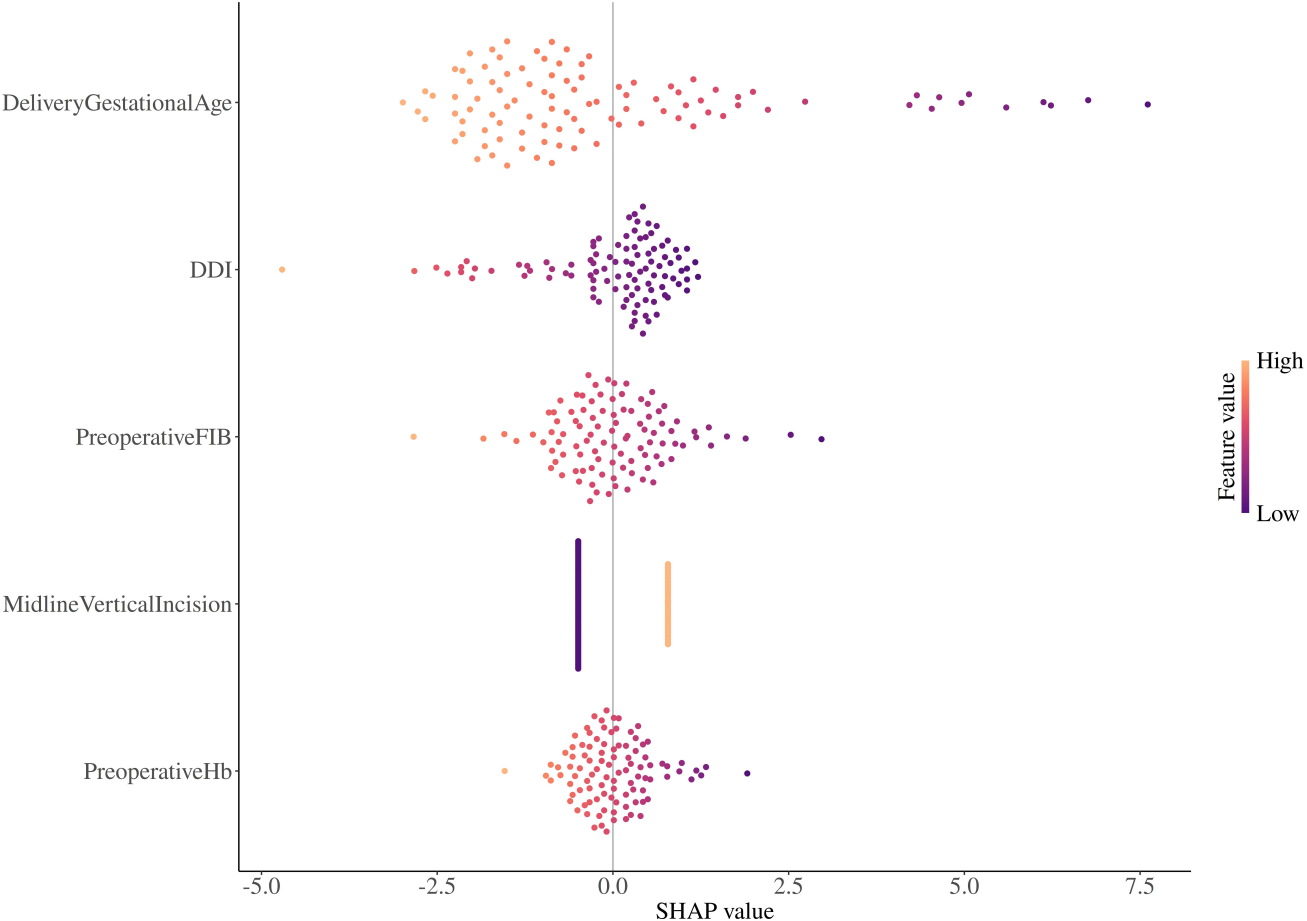
Beeswarm plot of SHAP values for predictors of the composite adverse outcome. Each point represents an individual patient. The position on the *x*-axis denotes the SHAP value (impact on the predicted log-odds of the outcome), and color corresponds to the patient’s value for that variable (blue: low, red: high). Variables are sorted by their mean absolute SHAP value (overall importance).

Variables significant on univariate analysis were included in a multivariate logistic regression model. Collinearity diagnostics revealed all variance inflation factors (VIFs) were below 2, indicating no substantial multicollinearity. The final model established midline vertical incision (OR = 3.59), shorter gestational age at delivery (OR = 0.90 per week), lower preoperative fibrinogen (OR = 0.46 per g/L), and a shorter DDI (OR per minute increase = 0.96, i.e., each minute decrease in DDI was associated with 4% higher odds of the composite outcome) as independent risk factors for the composite adverse outcome (all *P* < 0.05; Table 4). A forest plot summarizing these results is presented in Figure 4. To further interpret the model and quantify the contribution of each variable, we performed a SHAP analysis; the resulting beeswarm plot (Figure 5) confirmed that these same factors were the primary drivers of the model’s predictions, with younger gestational age and vertical incision having the largest impact.

### Predictive performance of the model and individual variables

**FIGURE 6:**
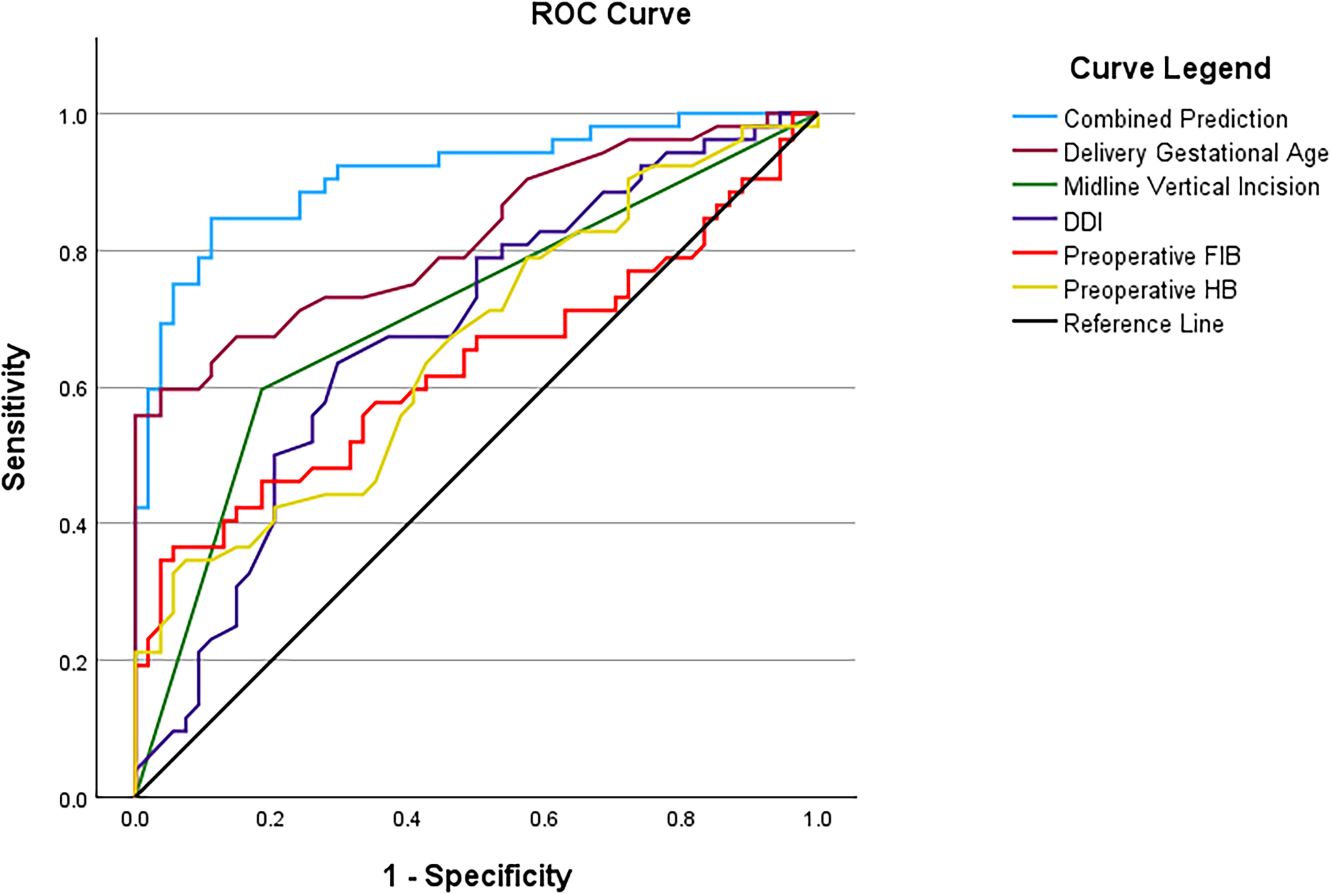
Receiver operating characteristic (ROC) curves for prediction of the composite adverse outcome.

The multivariate model’s predicted probability exhibited excellent discrimination for the composite adverse outcome (AUC = 0.911). In contrast, the AUCs for individual predictors were lower: 0.820 for gestational age at delivery, 0.679 for DDI, 0.660 for preoperative hemoglobin, and 0.629 for preoperative fibrinogen (Figure 6 and Table 5).

**TABLE 5:** Results of ROC curve analysis for predicting the composite adverse outcome.

	AUC (95% CI)	Optimal cutoff value	Sensitivity (%)	Specificity (%)	Youden’s index	*P*
Combined prediction	0.911 (0.855–0.967)	–	90.4	83.3	0.737	<0.001
Gestational age at delivery (Days)	0.820 (0.739–0.901)	269.5 days	73.1	63.0	0.361	<0.001
Midline vertical incision	0.705 (0.620–0.791)	–	59.6	81.5	0.411	<0.001
DDI	0.679 (0.576–0.781)	39.5 min	82.7	63.0	0.457	0.001
Preoperative Hb	0.660 (0.557–0.764)	107.5 g/L	34.6	92.6	0.272	0.002
Preoperative FIB	0.629 (0.519–0.738)	4.775 g/L	67.3	63.0	0.303	0.021

FIB, fibrinogen; Hb, hemoglobin; DDI, decision-to-delivery interval; Combined prediction: gestational age at delivery + midline vertical incision + DDI + preoperative Hb + preoperative FIB.

## Discussion

Placental abruption represents one of the most critical obstetric emergencies, in which the surgical approach for emergency cesarean delivery directly impacts maternal and neonatal prognoses. Utilizing a retrospective cohort design with PSM to control for baseline confounding, this study systematically compared maternal-neonatal outcomes between midline vertical and transverse incisions. Our principal findings robustly identified midline vertical incision as an independent predictor for the composite adverse outcome (OR 3.59, 95% CI 1.04–12.38). The clinical significance of this finding is powerfully underscored by the SHAP analysis (Figure 5), which ranked incision type as one of the two most impactful predictors in the model, demonstrating its substantial and consistent contribution to patient risk. This evidence elevates incision choice from a mere surgical preference to a key modifiable factor with a major influence on patient prognosis.

### Interpretation of findings and the trade-off between speed and morbidity

A paramount consideration in interpreting our results is the potential for confounding by indication. In this observational setting, the selection of a midline vertical incision was likely driven by the attending surgeon’s perception of extreme urgency or greater clinical severity–factors such as rapid fetal deterioration or concern for profound coagulopathy that are difficult to quantify and were not fully captured in our data. Despite propensity score matching, the persistently higher rates of uterine packing (51.2% vs. 16.9%) and general anesthesia in the vertical incision group suggest that residual channeling of more critical cases to this approach may have occurred. Thus, the strong independent association we observed between vertical incision and the composite adverse outcome (aOR = 3.59) may partly reflect the incision’s role as a marker of underlying clinical severity in addition to any direct physiological effect of the incision technique itself. Our findings, therefore, robustly demonstrate a significant clinical association that warrants attention, but they cannot definitively disentangle the effect of the incision from the effect of the clinical context that prompted its use.

Our findings challenge the deeply ingrained surgical heuristic of “go vertical to go fast” in the setting of placental abruption. The data suggest that the perceived urgency prompting a vertical incision may lead to a misplaced prioritization of skin-to-uterus time over overall procedural safety and tissue preservation. Importantly, DDI is often governed by system factors (anesthesia, readiness) rather than the minutes saved by the incision type itself (10–12). In abruption, where maternal physiology is already stressed by coagulopathy and hemorrhage, the additional tissue trauma and blood loss from a vertical incision may convert a time-critical situation into one burdened by compounded morbidity, without yielding the intended fetal benefit. Notably, the shorter DDI in the vertical group did not translate into improved fetal outcomes but was paradoxically associated with higher rates of neonatal asphyxia and NICU admission.

### Clinical implications, potential mechanisms, and comparison with previous studies

Within the context of the above caveat, the transverse incision group demonstrated significant advantages in operative time, estimated blood loss, need for uterine packing, and postoperative hospital stay. These findings align with the inherent characteristics of vertical incisions, which are prone to more extensive tissue damage and vascular compromise (13–15). Most notably, the incidence of surgical site complications was substantially higher in the midline vertical group (29.27%) compared to none in the transverse incision group, corroborating previous reports of increased wound complication risk with vertical incisions (16–18). Regarding neonatal outcomes, the higher rates of preterm birth, neonatal asphyxia, and NICU admission in the vertical incision group suggest that incision selection may indirectly adversely affect the compromised fetus, potentially through influences on uterine manipulation, placental perfusion, or the delivery pathway (19, 20). Notably, despite the potentially more complex nature of vertical incisions, we observed no significant intergroup differences in DDI or incision-to-delivery interval. This suggests that in placental abruption emergencies, DDI may be more constrained by preoperative processes, while incision type primarily influences procedural safety and tissue trauma extent during the delivery phase (21, 22).

The potential mechanisms can be interpreted from several perspectives: Anatomic considerations: midline vertical incisions more frequently damage abdominal wall vasculature and nerves while compromising fascial integrity, potentially increasing bleeding and impairing healing (15, 23). This is particularly detrimental in abruption patients who often exhibit a hyperfibrinolytic state and compromised healing capacity (24, 25). Physiological impacts: more extensive surgical trauma may exacerbate stress responses, impair utero-placental circulation, and impose secondary insults on the compromised fetus (26). Coagulation status: both our study and previous literature (27, 28) confirm that low preoperative fibrinogen represents a strong independent risk factor, underscoring the crucial role of coagulation reserve in managing abruption-related hemorrhage.

Our results corroborate existing observational studies that link vertical incisions to increased risks of uterine extension, transfusion, and composite surgical morbidity (29–32). Importantly, by focusing specifically on placental abruption, our study not only validates this association in a high-risk setting but also reveals its amplified effect–the vertical incision increased the risk of our composite adverse outcome (OR 3.59) beyond the typical effect sizes seen for single complications. This finding implies that the adverse impact of incision choice is particularly pronounced in this population. The comparable incidence of PPH between groups, a finding noted elsewhere (33–35), is plausibly explained by the dominant role of the underlying abruption-related coagulopathy and tissue injury in driving hemorrhage, potentially eclipsing the independent contribution of the incision (36–38).

Within the limitations of this observational study, our findings suggest that a transverse incision may be associated with more favorable outcomes for most patients undergoing emergency cesarean delivery for placental abruption, particularly in the absence of immediate life-threatening fetal bradycardia or anticipation of complex surgery. However, given the potential for confounding by indication, these findings should be interpreted with caution, and the midline vertical incision remains an important option for compelling clinical scenarios, such as profound and persistent fetal compromise unresponsive to resuscitation, or maternal hemodynamic collapse requiring rapid intra-abdominal access for hemorrhage control (39, 40). Ultimately, incision selection in this setting warrants individualized assessment.

### Limitations and future research

This study has several limitations that should be considered when interpreting our findings. As a retrospective single-center study, it remains susceptible to unmeasured confounding and selection bias despite the use of propensity score matching. The significantly lower gestational age and higher use of general anesthesia in the vertical group, even after matching, suggest that surgeons may have selected vertical incisions for more severe cases. The sample size, while balanced after matching (*n* = 106), is moderate. We lacked detailed data on abruption severity grading (e.g., extent of placental detachment), which could further refine risk stratification. As a single-center study from China, generalizability to other populations and healthcare settings may be limited. Despite these limitations, our findings align with anatomical and physiological rationale: vertical incisions are associated with greater tissue trauma and bleeding, which may be poorly tolerated in abruption patients with coagulopathy. The *E*-value analysis provides some reassurance that our core finding is relatively robust to unmeasured confounding.

Future prospective, multicenter studies are needed to establish causality and improve generalizability. Research priorities should include refined risk stratification by abruption severity, evaluation of long-term outcomes (e.g., uterine rupture risk in subsequent pregnancies, incisional hernia), and the development and validation of clinical decision-support tools that incorporate patient-specific factors and clinical acuity.

## Conclusion

In this single-center, retrospective propensity score-matched analysis of placental abruption, a midline vertical abdominal incision was independently associated with a shorter decision-to-delivery interval but also with a significantly higher risk of a composite adverse maternal-neonatal outcome, compared to a transverse incision. This robust association highlights a critical trade-off between procedural speed and morbidity in this emergency context. Given the observational design, these findings should be interpreted as identifying an important clinical association, the cause of which may be multifactorial and influenced by both the incision type and the underlying severity of the abruption.

## Data Availability

The original contributions presented in this study are included in this article/Supplementary material, further inquiries can be directed to the corresponding authors.

## Footnotes

1
www.medsta.cn/software
