# Supplementary material for: Vertical versus transverse abdominal incision in placental abruption: a propensity score-matched study on the trade-off between speed and maternal-neonatal safety
Source: Front Med (Lausanne). 2026 May 29;13:1804028. doi: 10.3389/fmed.2026.1804028 (PMC13260073; doi:10.3389/fmed.2026.1804028)
Supplement: Supplementary file 1 [file Table_1.docx]

STROBE Statement—Checklist of items that should be included in reports of ***cohort studies***

|  | Item No | Recommendation | Page No |
| --- | --- | --- | --- |
| **Title and abstract** | 1 | (*a*) Indicate the study’s design with a commonly used term in the title or the abstract | Title: “A Propensity Score-Matched Study” Abstract: “retrospective cohort study” |
|  |  | (*b*) Provide in the abstract an informative and balanced summary of what was done and what was found | Abstract (Objective, Methods, Results, Conclusions) – entire abstract |
| Introduction | | | |
| Background/rationale | 2 | Explain the scientific background and rationale for the investigation being reported | Introduction, first paragraph (lines 1–10) |
| Objectives | 3 | State specific objectives, including any prespecified hypotheses | Abstract (Objective) and Introduction, last paragraph (lines 15–20) |
| Methods | | | |
| Study design | 4 | Present key elements of study design early in the paper | Methods – Study Design and Ethical Approval (first paragraph) |
| Setting | 5 | Describe the setting, locations, and relevant dates, including periods of recruitment, exposure, follow-up, and data collection | Methods – Study Population and Data Collection: single center, January 2020 – December 2024 |
| Participants | 6 | (*a*) Give the eligibility criteria, and the sources and methods of selection of participants. Describe methods of follow-up | Methods – Inclusion and Exclusion Criteria (complete list); Data Collection (electronic medical records). Follow-up is in-hospital, described in outcome measures. |
|  |  | (*b*) For matched studies, give matching criteria and number of exposed and unexposed | Methods – Propensity Score Matching Methodology (covariates, 1:2 nearest neighbor, caliper 0.2 SD). Results – after matching: 41 vertical, 65 transverse. |
| Variables | 7 | Clearly define all outcomes, exposures, predictors, potential confounders, and effect modifiers. Give diagnostic criteria, if applicable | Methods – Outcome Measures (primary composite outcome), Data Collection (exposure = incision type; confounders listed). Grade 0 abruption defined in exclusion criteria. |
| Data sources/ measurement | 8* | For each variable of interest, give sources of data and details of methods of assessment (measurement). Describe comparability of assessment methods if there is more than one group | Methods – Data Collection (electronic medical records). Estimated blood loss method described (volumetric and gravimetric). Same methods for both groups. |
| Bias | 9 | Describe any efforts to address potential sources of bias | Methods – Propensity Score Matching (to balance confounders), Statistical Analysis (multivariate adjustment, SHAP). Discussion acknowledges confounding by indication. |
| Study size | 10 | Explain how the study size was arrived at | Methods – Sample Size Considerations: no a priori calculation; all eligible cases during study period (n=152, matched n=106). |
| Quantitative variables | 11 | Explain how quantitative variables were handled in the analyses. If applicable, describe which groupings were chosen and why | Methods – Statistical Analysis: continuous variables as mean±SD or median (IQR) based on distribution; groups compared with t-test or Mann-Whitney U. |
| Statistical methods | 12 | (*a*) Describe all statistical methods, including those used to control for confounding | Methods – Statistical Analysis: PSM, univariate/multivariate logistic regression, stepwise selection, VIF, SHAP, ROC with Youden index. |
|  |  | (*b*) Describe any methods used to examine subgroups and interactions | None pre-specified. SHAP analysis explored feature contributions but not formal interaction tests. |
|  |  | (*c*) Explain how missing data were addressed | Exclusion criteria: patients with missing medical records were excluded. No imputation performed. |
|  |  | (*d*) If applicable, explain how loss to follow-up was addressed | Not applicable (retrospective in‑hospital outcomes; no loss to follow‑up). |
|  |  | (*e*) Describe any sensitivity analyses | E-value analysis for unmeasured confounding mentioned in Discussion – Limitations (E-value = 6.64). |
| Results | | |  |
| Participants | 13* | (a) Report numbers of individuals at each stage of study—eg numbers potentially eligible, examined for eligibility, confirmed eligible, included in the study, completing follow-up, and analysed | Results – Figure 1 (flowchart). Text: 152 initially, 106 after PSM (41 vertical, 65 transverse). |
|  |  | (b) Give reasons for non-participation at each stage | Figure 1 and Exclusion criteria (e.g., prior CS, major comorbidities, missing data). |
|  |  | (c) Consider use of a flow diagram | Figure 1 is a flow diagram. |
| Descriptive data | 14* | (a) Give characteristics of study participants (eg demographic, clinical, social) and information on exposures and potential confounders | Table 1 (baseline characteristics before/after PSM), Table 2 (surgical/laboratory parameters). |
|  |  | (b) Indicate number of participants with missing data for each variable of interest | No missing data after applying exclusion criteria (stated in Methods). |
|  |  | (c) Summarise follow-up time (eg, average and total amount) | Not applicable (only in‑hospital outcomes; no longitudinal follow‑up). |
| Outcome data | 15* | Report numbers of outcome events or summary measures over time | Table 3 (maternal and neonatal outcomes). Primary composite outcome: 32.3% transverse vs. 75.6% vertical. |

| Main results | 16 | (*a*) Give unadjusted estimates and, if applicable, confounder-adjusted estimates and their precision (eg, 95% confidence interval). Make clear which confounders were adjusted for and why they were included | Table 4: univariate (crude OR) and multivariate (adjusted OR). Adjusted for gestational age, fibrinogen, DDI, incision type. Covariates chosen based on clinical knowledge and univariate p<0.10. |
| --- | --- | --- | --- |
|  |  | (*b*) Report category boundaries when continuous variables were categorized | Continuous variables were not categorized in regression. ROC analysis (Table 5) provides optimal cutoffs (e.g., DDI ≤39.5 min, gestational age ≤269.5 days). |
|  |  | (*c*) If relevant, consider translating estimates of relative risk into absolute risk for a meaningful time period | Not done (OR reported; absolute risk can be derived from event rates in Table 3). |
| Other analyses | 17 | Report other analyses done—eg analyses of subgroups and interactions, and sensitivity analyses | SHAP analysis (Figure 5) to quantify feature importance; ROC curves (Figure 6) for model discrimination. Sensitivity analysis: E-value in Discussion. |
| Discussion | | | |
| Key results | 18 | Summarise key results with reference to study objectives | Discussion, first paragraph (vertical incision associated with faster DDI but higher composite adverse outcome; aOR=3.59). |
| Limitations | 19 | Discuss limitations of the study, taking into account sources of potential bias or imprecision. Discuss both direction and magnitude of any potential bias | Discussion – Study Limitations (single-center, retrospective, residual confounding, sample size, lack of abruption severity grading, generalizability). E-value discussed. |
| Interpretation | 20 | Give a cautious overall interpretation of results considering objectives, limitations, multiplicity of analyses, results from similar studies, and other relevant evidence | Discussion – Interpretation of Findings... and Conclusion (trade-off between speed and morbidity; confounding by indication possible; individualized assessment). |
| Generalisability | 21 | Discuss the generalisability (external validity) of the study results | Discussion – Limitations and Future Research (single-center from China; may not generalize to other populations/settings). |
| Other information | | | |
| Funding | 22 | Give the source of funding and the role of the funders for the present study and, if applicable, for the original study on which the present article is based | Funding section: Self-funded Research Project of the Health Commission of Guangxi Zhuang Autonomous Region (grant Z‑A20250900). Funders had no role in study design, data collection, analysis, or manuscript preparation. |

*Give information separately for exposed and unexposed groups.

**Note:** An Explanation and Elaboration article discusses each checklist item and gives methodological background and published examples of transparent reporting. The STROBE checklist is best used in conjunction with this article (freely available on the Web sites of PLoS Medicine at http://www.plosmedicine.org/, Annals of Internal Medicine at http://www.annals.org/, and Epidemiology at http://www.epidem.com/). Information on the STROBE Initiative is available at http://www.strobe-statement.org.
